# Hypothalamic Astrocytes as a Specialized and Responsive Cell Population in Obesity

**DOI:** 10.3390/ijms22126176

**Published:** 2021-06-08

**Authors:** Ismael González-García, Cristina García-Cáceres

**Affiliations:** 1 Institute for Diabetes and Obesity, Helmholtz Diabetes Center at Helmholtz Zentrum München, 85764 Neuherberg, Germany; garcia-caceres@helmholtz-muenchen.de; 2 German Center for Diabetes Research (DZD), 85764 Neuherberg, Germany; 3 Medizinische Klinik und Poliklinik IV, Klinikum der Universität, Ludwig-Maximilians-Universität München, D-80336 Munich, Germany

**Keywords:** astrocytes, neurons, hypothalamus, obesity

## Abstract

Astrocytes are a type of glial cell anatomically and functionally integrated into the neuronal regulatory circuits for the neuroendocrine control of metabolism. Being functional integral compounds of synapses, astrocytes are actively involved in the physiological regulatory aspects of metabolic control, but also in the pathological processes that link neuronal dysfunction and obesity. Between brain areas, the hypothalamus harbors specialized functional circuits that seem selectively vulnerable to metabolic damage, undergoing early cellular rearrangements which are thought to be at the core of the pathogenesis of diet-induced obesity. Such changes in the hypothalamic brain region consist of a rise in proinflammatory cytokines, the presence of a reactive phenotype in astrocytes and microglia, alterations in the cytoarchitecture and synaptology of hypothalamic circuits, and angiogenesis, a phenomenon that cannot be found elsewhere in the brain. Increasing evidence points to the direct involvement of hypothalamic astrocytes in such early metabolic disturbances, thus moving the study of these glial cells to the forefront of obesity research. Here we provide a comprehensive review of the most relevant findings of molecular and pathophysiological mechanisms by which hypothalamic astrocytes might be involved in the pathogenesis of obesity.

## 1. The Hypothalamus: Integrator of Neuroendocrine and Systemic Metabolic Homeostasis

The organization and cellular components of mammalian neuroendocrine systems are highly evolutionarily conserved and require circuits precisely tuned throughout life. The central nervous system (CNS) senses and processes local and peripheral afferent metabolic signals emanating from circulation (hormones, nutrients), and translates information from sensory nerves into physiological responses regulating feeding behavior and energy expenditure [1,2,3]. The identification of the hypothalamus as a metabolic center arose from pioneered observations during the mid-20th century. These seminal studies demonstrated first that inflicting lesions or the presence of tumors in specific hypothalamic brain areas elicited either hyperphagic obesity or severe anorexia [4]. Subsequently, the advances in technologies and knowledge allowed for further confirmation that the key role of the hypothalamus is its ability to integrate and process metabolic cues of endocrine and autonomic nervous system origins; thus, positioning this brain area at center stage in the regulation of body weight and energy balance.

The hypothalamus is structured and organized into different nuclei with the arcuate nucleus (ARC) having been more deeply studied for containing specialized neuronal populations. These neurons are vastly interconnected between themselves and with other extra-hypothalamic areas, which allows for a high intercommunication and redundancy in the homeostatic mechanisms [5]. The ARC is located at the base of the hypothalamus in close proximity to the median eminence (ME) and it is there where two neuronal populations well-known for being fundamental for whole-body energy regulation reside: Neuropeptide Y and agouti-related peptide (NPY/AgRP) expressing neurons whose activation exert orexigenic effects and reduce energy expenditure, and proopiomelanocortin (POMC) expressing neurons with opposing effects on feeding behavior and energy expenditure compared to NPY/AgRP neurons [1]. Both sets of neurons express receptors for peripheral endocrine inputs (e.g., leptin, insulin, GLP1, free fatty acids, and ghrelin), but also for central-derived signals (e.g., NPY, GABA, serotonin, and melanocortin), and the overall result of the integration and processing of these signals is to influence the wiring and flexibility of neuronal circuits for the homeostatic maintenance of energy balance. Thus, the connectivity of these ARC neuronal circuits is flexible, varying depending on the energy storage and nutritional state of the entire organism. Such adaptive plasticity of neuronal connectivity requires the chemical and physical association and cooperation of adjacent glial cells, which are active units in the regulation of synaptic function. 

In the last few years, hypothalamic astrocytes have started to draw some attention in the neuroendocrine field for acting as hypothalamic functioning regulators, particularly for their role in the central control of metabolism. Indeed, several findings emphasize the crucial involvement of astrocytes for their functional interactions with specific ARC-resident neuronal populations [6,7,8,9,10] together forming one unique functional circuit in metabolic regulation. The advances in neuroscience and molecular biological technologies designed for targeting non-neuronal cells are allowing us to advance current knowledge by providing novel concepts about the functional contribution of astrocytes in the physiological regulation of food intake and energy homeostasis.

## 2. Hypothalamic Astrocytes: A Specialized Population in Regulating Metabolism

The hypothalamus, in particular its ventral border where the ARC is located, is beneficially situated adjacent to the ME, a circumventricular organ with a particular angioarchitecture (i.e., fenestrated microvessels which lack blood brain barrier) positioning it as a more metabolically sensitive brain center over others in the CNS [11,12,13]. Such high and rapid responsiveness to metabolic disturbances is also due to being highly-enriched in a wide-array of metabolic receptors and transporters acting as primary sensors and responders to nutrients and endocrine cues emanating from the bloodstream [14,15,16,17]. Additionally, emerging insights highlight that hypothalamic astrocytes, tightly connected to ARC-resident neurons and blood vessels [18], are strategically situated to survey the organism’s metabolic status and to, in turn, remodel local vascular beds thereby controlling the selective access of some circulating factors into the brain [19]. Earlier studies have also indicated that hypothalamic astrocytes are particularly and primarily affected in response to metabolic damage induced by hypercaloric diets [20], positioning these glial cells as the center of attention for obesity research. In a more physiological context though, current literature underlines the role of astrocytes in the control of metabolism by their role of nutrient and hormone sensing regulation, and via the release of gliotransmitters in crosstalk with neurons.

### 2.1. Hypothalamic Astrocytes: In Bidirectional Tuning with Neurons for Hypothalamic Feeding Control

In recent years, several studies based on Designer Receptor-Exclusively Activated by Designed Drug (DREADD) have revealed that changes in astrocyte activity can impact the feeding behavior response to leptin and ghrelin. In 2015, a study led by Yang and colleagues showed that activation of astrocytes within the mediobasal hypothalamus (MBH) leads to reduced feeding by inhibiting AgRP neurons via adenosine A1 receptors, both in basal conditions and after ghrelin-evoked feeding. Moreover, pharmacogenetic activation of MBH-astrocytes increases leptin’s anorectic effect [6]. This study proposed a mechanism by which astrocytes participate in food intake regulation through the release of adenosine, a gliotransmitter known by its inhibitory action on both pre-and post- synaptic neurons [21], leading to the suppression of AgRP neuronal activity and its orexigenic action. Contrary to this, a later study showed opposing results, observing an increase in AgRP neuronal stimulation and food intake after chemogenetic activation of astrocytes [7]. These discrepancies between studies could be explained by the heterogeneous nature of the glia–neuronal interconnections in the hypothalamic feeding circuits where their connectivity and plasticity can vary depending on health (also influenced by sexual hormones and age) and metabolic status of the animal. Therefore, small alterations in the experimental paradigm such as time, last meal, environment, the anatomical location, and number of hypothalamic astrocytes chemogenetically manipulated could shift the circuit specific manipulation for controlling feeding and thus explain the diverse result observed. A recent study has pointed out the essentiality of a regulatory role of AgRP neurons over adjacent astrocytes for dynamic fine-tuned adaptations of their activity as part of homeostatic control of feeding [10]. This study has shown that chemogenetic or ghrelin-evoked activation of AgRP neurons promotes the release of GABA to increase astrocytic endfeet coverage on the membrane of AgRP neurons and triggers prostaglandin E2 release to subsequently increase the excitability of AgRP neurons via EP2 receptors. These findings support a dynamic and bidirectional communication between AgRP neurons and astrocytes for a feed-forward autoactivation loop of hunger circuits. Other studies have also reported that a gliopeptide Acyl-CoA–binding protein–derived (ACBP-derived) and its product octadecaneuropeptide (ODN) secreted by astrocytes is important for hypothalamic astrocyte–neuron tuning in the regulation of energy balance. In particular, they showed that pan-brain deletion of the ACBP gene in astrocytes promotes diet-induced hyperphagia and obesity in mice. Mechanistically, they proposed a “gliogenic” endozepine released mechanism based on the ACBP–ODN signaling in ARC astrocytes to influence the activity of POMC neurons to reduce feeding and weight gain [9].

### 2.2. Hypothalamic Astrocytes: Nutrient and Hormone Sensing-Dependent Regulation

***Lipid sensing.*** Astrocytes uptake lipids and store them in the form of droplets via several lipid transporters, including lipoprotein lipase (LPL), which is essential for the control of cellular lipid storage in the brain. When *lpl* is postnatally deleted in astrocytes, mice develop an aggravated obesogenic phenotype characterized by increased body weight gain and glucose intolerance in response to a high fat diet [22]. Mechanistically, it was found that these mice exhibited increased ceramide accumulation, specifically in hypothalamic neurons, a phenomenon observed in neurodegenerative processes induced by lipotoxicity [13,23]. In vitro studies have reported that hypothalamic astrocytes accumulate lipid droplets under saturated fatty acid-rich conditions, adopting a reactive inflammatory profile and eliciting chemotactic activity of microglia to produce inflammatory mediators [24]. Other in vitro work in human astrocytes has also pointed out a direct link between a disruption of lipid metabolism in astrocytes and insulin resistance [25]; however, the use in this case of cultured astrocytes would require additional confirmation for its relevance at hypothalamic level.

***Ketone body sensing.*** Besides lipid homeostasis function, astrocytes play a key role in fatty acid oxidation by synthesizing the majority of ketone bodies (KB) in the CNS. Studies in mice have shown that short-term exposure to a hypercaloric diet (HFD) is associated with higher levels of astrocyte-derived KB in the hypothalamus and an acute decrease in food intake. Accordingly, pharmacological inhibition of ketone synthesis blunts HFD-induced anorexia, supporting a model where astrocytes regulate fatty acids and KB levels in the hypothalamus as a lipid sensing mechanism, which is disrupted in leptin resistant obese rats [26]. Further, in vitro studies with the ketone body β-hydroxybutyrate have shown the direct effect of KBs on lowering the consumption of glucose and higher ability of mitochondria to metabolize pyruvate in mouse astrocytes, and consequently affecting the delivery of glucose and lactate to neurons [27]. Overall, these in vitro and in vivo findings indicate that astrocytes play a key role in maintaining a tight regulation of hypothalamic lipid homeostasis necessary for correct metabolic control. Further studies will need to address in detail how the lipogenic/lipolytic state of hypothalamic astrocytes is able to determine its cellular bioenergetics and communication with the surrounding neurons for the regulation of metabolism.

***Glucose sensing***. The brain has high energetic requirements, with glucose being its preferred fuel. However, glucose or energy storage in the brain is rather limited and restricted to astrocytes that, unlike neurons, are able to store glucose in the form of glycogen deposits in order to energetically support themselves through its lactate-shuttle transport [28,29]. It must be noted though that other authors have raised some questions about whether lactate oxidation primarily occurs in neurons and therefore the lactate-shuttle transport model probably needs further validation [30]. Independent of this role of astrocytes in energy storing, they also behave as glucose sensors. One of the first pieces of evidence of astrocyte involvement in central glucose sensing was provided by studies based on glucose transporter 2 (GLUT2) interrogation, which demonstrated that its inhibition in the ARC impaired glucose sensing by inhibiting the insulin response in response to intra-carotid glucose injection [31]. Interestingly, it was described that GLUT2 is mainly expressed in astrocytes instead of neurons within the hypothalamus [32,33,34]. Later studies using *glut2* global knockout (KO) mice confirmed the relevance of astrocytes in central glucose sensing. The mice globally lacking GLUT2 exhibited elevated blood glucagon levels and a decreased glucagon secretion in response to fluctuations in systemic glucose, a phenotype which illustrates that GLUT2 is essential for the counterregulatory response to hypoglycemia. Further, transgenesis-induced GLUT2 rescue specifically in astrocytes was able to restore glucagon secretion [35]. However, these last effects were specifically observed in astrocytes located in the brainstem, a relevant brain region regarding astrocyte glucose sensing and metabolism [36,37,38]. Glucose sensing is also a mechanism involved in the control of food intake, and thus the effects derived from hypothalamic astrocytes affect the way energy balance is regulated. In this regard, a different study showed that transgenic mice with inhibited GLUT2-mediated glucose detection augmented daily food intake by a mechanism that increased the meal size, but not the number of meals [39]. GLUT2 is located not only in astrocytes [32,35,40], but also in tanycytes and neurons [41,42]; therefore, these findings cannot be attributed solely to the astrocytic glucose transporter. In parallel, astrocytes located within the hypothalamus were also associated with the glucose sensing mechanism via GLUT1 in streptozotocin (STZ)-treated rats. Virogenetic overexpression of GLUT1 in hypothalamic astrocytes of STZ-diabetic rats with reduced GLUT1 normalizes circulating glucose levels and restores hypothalamic glucose sensing in rats, namely it rescues glucose production measured in intrahypothalamic clamp studies [43]. These findings support hypothalamic astrocytes as being essential components of the glucose sensing mechanism.

***Insulin sensing***. Insulin receptor (IR) expressed in hypothalamic astrocytes has been proven critical for the entry of glucose into the brain and for the modulation of glucose metabolism within the brain. Indeed, mice with postnatal *ir* knockout in astrocytes develop a defective anorectic response to glucose and an altered glucose homeostasis [8,44]. Despite this, genetically ablated mice lack IR in the whole brain, the phenotype was reproduced under a virogenetic deletion approach, thus proving that astrocytes within the hypothalamus were sufficient to induce the changes in glucose homeostasis. Moreover, we observed that the glucose-evoked reduction in neuronal activation occurred specifically in the hypothalamus and particularly in POMC neurons, thus reinforcing the special relevance of astrocytes located in this brain region [8]. Subsequent in vitro studies confirmed that loss of IR in astrocytes downregulates the expression of GLUT1 [45], while insulin-like growth factor 1 receptor (IGF1R) cooperates together with insulin in order to stimulate glucose uptake by astrocytes [46]. Importantly, in vivo experiments have also shown that IR ablation in astrocytes induces a compensatory upregulation of IGF1 receptor [45] and therefore, the fact that these two receptors have high homology and convergent signaling pathways underlines the importance of understanding their dual functional role in astrocytes.

***Leptin sensing.*** Initial studies in agouti viable yellow (Avy) mice, showed that leptin receptor (LepR) in astrocytes may play a role in leptin transport across the BBB and in the regulation of adult-onset obesity of these animals [47,48]. Molecular analysis of the brain in the same model, in addition to HFD-fed mice, showed that obesity rapidly increases LepR expression in astrocytes where it then influences the leptin-induced calcium signaling in astrocytes [49]. Later, additional studies showed that central and chronic leptin administration modifies hypothalamic astrocyte morphology and glutamate transporters [50,51]. The subsequent generation of specific astrocyte LepR ablation mouse models confirmed these initial findings. In this regard, constitutive astrocyte-specific *lepr* (KO) mice did not develop an altered phenotype in terms of energy balance, likely due to a compensatory mechanism [52]. However, inducible astrocyte-specific *lepr* KO mice exhibited a lack of proper anorectic response to leptin and showed an exacerbated refeeding after fasting. Interestingly, both physiological alterations were associated with changes in POMC neurons (i.e., lower activation after leptin treatment) and AgRP neurons (i.e., overactivation during the refeeding phase) [53]. Overall, these findings support the existence and functional relevance of LepR in astrocytes. In agreement with previous reports, our work has recently confirmed the specific presence of the truncated LepR_a_ in fluorescence-activated cell sorting (FACS)-isolated hypothalamic astrocytes [19].

***GLP1 sensing***. Recent studies have highlighted the role of astrocytic glucagon-like peptide-1 (GLP-1) signaling in the regulation of brain glucose uptake [54]. When GLP-1R was postnatally and genetically ablated in hypothalamic astrocytes, mice displayed an increased brain glucose uptake, which was associated with an improvement in memory formation and systemic glucose response [54]. Further evidence suggested that these effects are mediated through fibroblast growth factor (FGF)-21 action, given that neutralization of central FGF21, as well as astrocyte-specific ablation of FGF21, abrogated the improvements in glucose metabolism observed in the astrocyte-specific *glp-1r* knockout mice [54].

## 3. Hypothalamic Astrocytes in the Physiopathological Development of Obesity

Based on human genome-wide association studies (GWAS) from obese patients, it has recently emerged that the brain, in particular the hypothalamus, controls most aspects of systemic metabolism over other classically described peripheral endocrine axes, suggesting that obesity is largely a brain disease [55]. Interestingly, clinical studies have reported that being obese is associated with brain atrophy; describing alterations in food reward-regulating circuits [56], nutrient and hormone sensing-dependent neuronal activity [57,58], hypothalamic gliosis [20,59,60,61], and angiopathy [62], all being signs of neuronal dysfunction and aging [63]. Most of these changes have been reported to originate from the hypothalamus, which is considered a selective vulnerable area in diet-induced obesity and has been suggested as a hallmark of neuronal dysfunction associated with the initiation and progression of this pathology [64,65]. In fact, it is thought that obesity-induced hypothalamic dysfunction emerges from aberrant glia responses (astrocyte and microglia) inducing impairments in the connectivity and activity (responsiveness) of ARC-resident neuronal circuits responsible for orchestrating metabolic control and contributing to the aggravation of this pathology [64,65,66,67]. Despite the relative contribution of each glial cell type [68] and their crosstalk [69] to obesity pathophysiology being a current topic of research in this section, we will focus in the astrocyte-related mechanisms.

### 3.1. Inflammatory-Related Mechanisms

One of the most established pathophysiological mechanisms to explain obesity development is the inflammatory and oxidative stress hypothesis, mediated through different pathways, occurring in different endocrine tissues, including in the hypothalamus. Early studies pointed out that rich caloric diets induce a low-grade inflammation within the hypothalamus characterized by a rapid rise in cytokines [70], gliosis, and metabolic stress leading to dysfunction of hypothalamic circuits and consequent obesity [20]. Moreover, hypothalamic inflammation has also been associated with the development of central resistance to endocrine signals before changes in body weight [20,70,71]. Indeed, hypothalamic inflammatory processes are tightly linked with hormone resistance and obesity. This is supported by studies reporting that amelioration of hypothalamic inflammatory processes i.e., a blunted tumor necrosis factor alpha (TNFα) signaling, an ablated suppressor of cytokine signaling-3 (Socs3), and the disruption of hypothalamic-specific interleukin (IL)-6) led to rescue of both leptin and insulin signaling and protection from the metabolic alterations induced by HFD [72,73,74]. Several pathways have been proposed as mediators of hypothalamic inflammatory responses in diet-induced obesity. Notably, some studies pointed out that the IkB kinase-β (IKKβ)/nuclear factor κB (NF-κB) pathway mediates the expression of several pro-inflammatory genes within the hypothalamus, including cytokines and chemokines [75], and leads to endoplasmic reticulum (ER) stress, a cellular process characterized by the accumulation of misfolded or unfolded proteins in the ER lumen [76,77]. Both signaling pathways are actually interconnected, and it was shown that HFD leads to increased ER stress and consequently activates IKKβ–NF-κB signaling in the hypothalamus, producing the altered response to hormones and ultimately leading to metabolic homeostasis dysregulation. 

### 3.2. Reactive Astrogliosis in Obesity

This term, which recently reached a consensus agreement on its definition, together with “astrocyte reactivity” or “reactive astrocytes” [78], entails the morphological and/or functional changes seen in astrocytes responding to healing processes or pathophysiology [79]. Seminal studies led by Horvath and colleagues reported that diet-induced obesity in mice also induces reactive astrogliosis in the hypothalamus [18]. The ultrastructural analyses of these brains also showed that, concomitant with the findings, there were changes in the physical interactions of astrocytes with endothelial cells and neurons causing alterations of the cytoarchitecture and synaptology of hypothalamic circuits [18]. Surprisingly, these astrocytic morphological changes, as well as the increased cytokine production in the hypothalamus, occurred before any sign of systemic inflammation or body weight gain [20]. HFD-induced reactive astrogliosis is characterized by an upregulation of the structural protein glial fibrillary acidic protein (GFAP), promotion of a pro-inflammatory phenotype (producing and releasing cytokine markers), and the acquisition of a hypertrophic morphology, but without proliferation-unlike what was observed in other brain pathologies. Remarkably, a recent study has found that GFAP gene induction occurs very early, and even one hour of HFD exposure is sufficient to find a significant change. That short exposure interferes also in the neuropeptide and an inflammatory-like gene responses exerted by the chemogenetic activation of astrocytes [80].

Blocking inflammatory-induced astrocyte activation via expression of a dominant-negative form of the NFκB inhibitor, IκBα, in GFAP^+^ astrocytes in a doxycycline-inducible manner showed an inhibition of HFD-induced reactive astrogliosis in the hypothalamus coupled with a promotion of hyperphagia, even 24 h following the dietary switch [81]. These findings suggest that the NFκB-IκBα pathway in astrocytes controls HFD-induced reactive astrogliosis illustrating acute morphological adaptations in astrocytes for precisely controlling food intake [81]. Of note, astrocyte changes described here were found in the MBH; the genetic mouse model was generated modifying all GFAP^+^ astrocytes in the CNS and therefore an indirect effect via non-hypothalamic regions cannot be ruled out. Douglass and colleagues found that postnatal ablation of IKKβ specifically in astrocytes after HFD exposure has a protective effect on weight gain mediated through a reduction in food intake and an increase in energy expenditure, which was also accompanied by an improved glucose tolerance and insulin sensitivity. Importantly, both hypothalamic inflammation and reactive astrogliosis were diminished in HFD-fed IKKβKO mice [82]. These findings were similarly supported by other authors showing that astrocytic IKKβ/NF-κB upregulation is a key signaling pathway during overnutrition, which mediates some of the early-onset effects of obesity. Mechanistically, astrocytic activation via IKKβ/NF-κB seems to modify astrocytic morphology (i.e., decreased astrocytic process density), a downstream regulation of hypothalamic extracellular GABA levels, and BDNF expression within the hypothalamus [83]. That being said, hypothalamic inflammation and reactive astrogliosis have been used as generic terms to describe a variety of morphological and molecular changes occurring under the effects of high fat diet exposure, sometimes describing phenotypes under the same phenomena, which only partly fulfill the molecular and cellular characteristics of the other phenotypes reported. In this regard, there is a clear demand for neuroscientists to agree on a clear and meaningful nomenclature, which precisely describes the morphological and functional readouts to evaluate.

Some studies indicate that dietary factors such as fat are determinant triggers to the hypothalamic inflammatory response and the changes induced in glial cells. In vitro studies have pointed out that saturated fatty acids (i.e., palmitic, lauric, and stearic acid) induce the release of TNF-α and IL-6 from cultured astrocytes [84]. Mechanistically, saturated fatty acids require toll-like receptor 4 (TLR4) to induce cytokine release, while the pharmacological inhibition of p38 or p42/44 MAPK pathways precludes the pro-inflammatory actions of the saturated fatty acids [84]. In agreement with these findings, TLR2/4-deficient mice were protected from HFD-induced weight gain as well as glucose intolerance and insulin resistance. Moreover, C3H/HeJ/TLR2-deficient mice also showed a decreased expression of IL-6 in both basal and insulin-stimulated states. Following that, treatment with an IL-6-neutralizing antibody led to a rescue of insulin sensitivity in HFD-fed mice despite no changes being found in body weight [85]. Gao and colleagues found that while reactive astrogliosis was mainly a response to the fat content of the diet, it is the combination of sucrose and fat consumption that are responsible for stimulating some other aspects of hypothalamic inflammatory response, such as microgliosis and angiogenesis [86]. Lastly, other studies have highlighted that the astrocytes’ response to develop an HFD-induced hypertrophic phenotype can vary depending on the age of the mice. Specifically, Lemus and colleagues observed that aged mice showed a higher GFAP expression in basal conditions than younger ones, which can affect the visualization of differences between mice fed with chow diet and HFD at a certain age [87]. Aside from diet-induced obesity, other studies have also reported the presence of reactive astrogliosis in monogenetic genetic obese mouse models [47,49,88], or by maternal dietary influence during the fetal and early stages of life of the offspring [51]. Therefore, a careful interpretation has to be taken about the context in which reactive astrogliosis is evaluated because the molecular pathways entailed and functional implications in obesity development could vary depending on the nature of the trigger (e.g., dietary factor, aberrant metabolic, or hormone inputs), the brain areas involved, the status of surrounding cellular compounds (microgliosis and neuronal connectivity), as well as other relevant parameters, such as health state and age of the animal that could interfere with the resulting outcomes and conclusions.

### 3.3. Alterations in the Astroglia-Vascular Interface in Obesity

**Figure 1:**
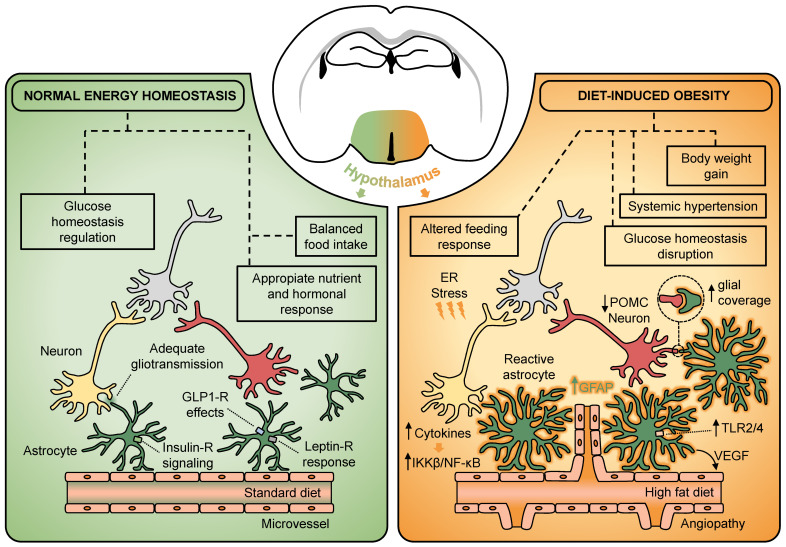
Hypothalamic astrocytes participate in the regulation of metabolic homeostasis and act as key players in the development of diet-induced obesity. Astrocytes located in the hypothalamus express several endocrine receptors such as, insulin, leptin, and GLP-1. The action of hormones and pharmacological analogues on hypothalamic astrocytes has been described to influence several aspects of normal energy homeostasis. Under energy-dense diet exposure, the hypothalamus experiences a low-grade inflammation characterized by a rapid rise in cytokines, which trigger downstream pathways such as the IKKβ/NF-κB and TLR2/4. This also leads to increased hypothalamic ER stress, which seems to interfere with the adequate response to hormones. Together, there is an HFD-reactive astrogliosis characterized by an overexpression of GFAP, the acquisition of a hypertrophic morphology, and a synaptic input reorganization of the melanocortin system, which may suggest a direct involvement of hypothalamic astrocytes in the pathogenesis of diet-induced obesity. At vascular level, obesogenic diets trigger astroglial VEGF-driving angiogenesis in the hypothalamus to promote the development of systemic hypertension. ER, endoplasmic reticulum; IKKβ, IkB kinase-β; GFAP, glial fibrillary acidic protein; GLP-1, glucagon-like peptide-1; NF-κB, nuclear factor κB; POMC, proopiomelanocortin; TLR2/4, toll-like receptor 2/4; VEGF, vascular endothelial growth factor.

HFD-induced reactive astrogliosis has been detected in specific hypothalamic regions, in particular, those ones close to the third ventricle, such as the MBH [18,64,88], which have a higher accessibility of circulating factors and are partly placed out of the BBB [88]. Indeed, HFD-fed mice show a significant increase in blood vessel length and density in the MBH, as well as a lack of BBB integrity [62]. Interestingly, human diabetic patients exhibited an increased number of arteries and arterioles in the hypothalamic infundibular nuclei, suggesting a potential link between hypothalamic vascular remodeling and the development of metabolic diseases in humans [62]. A recent study by our group has further dissected the pathogenic processes leading to such impairments, uncovering the role of hypothalamic astrocytes in promoting hypothalamic microangiopathy and the systemic arterial hypertension associated with obesity. By using virus-mediated gene transfer, it was found that astroglial vascular endothelial growth factor (VEGF) in the hypothalamus exerts significant effects on systemic blood pressure control via tuning sympathetic outflow. These new findings supporting a novel mechanism with hypothalamic angiopathies at its center are important as they could explain how obesity-associated hyperleptinemia rather than adiposity causes hypertension [19] (Figure 1).

## 4. Outlook

The hypothalamus, with its different nuclei, is a good example of how different neuronal types and connections translate to functional specialized roles. Given the complex nature of hypothalamic cytoarchitecture where circuits that play opposing roles in the control of metabolism are tightly intermingled, it will not be surprising that the study of astrocytes in the control of metabolism should be approached to specifically address the precise anatomical location of the circuits and the interaction among them. To date, several studies have contributed to understanding the functional relevance of astrocytes in the development of obesity; however, a big gap is still present on how these alterations affect the neural networks in which astrocytes are embedded. Thus, the idea of a disruption in the astrocyte-neuron communication within the hypothalamus seems a plausible, yet poorly investigated, hypothesis that could contribute to the pathophysiology of obesity. Research on how astrocyte–neuron communication is altered by exposure to obesogenic insults will allow a better understanding of the hypothalamic alterations that occur in this context.

Another important factor linked to the regional specialization is astrocyte heterogeneity. Regardless of the classical morphological taxonomy (i.e., fibrous and protoplasmic astrocytes), the specific molecular markers label a wide range of astrocyte subpopulations, which could also indicate a complex array of functional actions. In the upcoming years, an important topic to address will be the relative contribution of each subpopulation to the development of obesity. Taking advantage of the novel technological advances in the study of cell- and synapse-specific functionality by using combined two-photon imaging, patch-clamp recordings, cellular mapping of transcriptomic profiles in the tissue context using single-cell RNA sequencing, and new in situ hybridization technologies. In combination, researchers will be able to understand the tissue structure and function on a single astrocyte level, helping to identify specific hypothalamic astrocyte subpopulations, which might be critical for the pathophysiology and development of obesity.

## Data Availability

Not applicable.
